# Materno-Fetal Transmission of Human Immune Deficiency Virus

**DOI:** 10.1155/S1064744997000185

**Published:** 1997

**Authors:** Axel Schäfer

**Affiliations:** 1 Homboldt-Universität zu Berlin Berlin Germany; 2 Frauenklinik und Polikinik Augustenburger Platz 1 Berlin 13353 Germany

## Abstract

Mother-to-child transmission of human immune deficiency virus (HIV) is a multifactorial event highly associated with advanced maternal HIV disease and obstetric incidents taking place during parturition. Thus, various approaches to prevention may be beneficial. Although the time and the route of materno-fetal HIV transmission are still not sufficiently clear, much speaks in favor of a late HIV transmission, most probably taking place during parturition or the phase before the delivery. The fetus is remarkably protected by the placenta and the intact fetal membranes against many viral infections during gestation. These conditions change at parturition and the chance for a transition of HIV-infected carrier cells or virus into the fetal compartment increases. Proinflammatory cytokines secreted at the materno-fetal interface accumulate in amniotic fluid and may chemoattract and stimulate potentially HIV-infected immunocytes. After rupture of membranes, maternal cells of the decidua are directly exposed to the amniotic fluid. Aside from the contamination of the fetal skin at vaginal delivery as a debatable route of infection, blood-to-blood contacts and the fetal swallowing of contaminated amniotic fluid may be the major path of fetal HIV infection. For the fetal prophylaxis of an intrauterine infection, the application of zidovudine is recommended. However, cesarian section before the onset of labor leads also to a diminution of the transmission rate. As the transmission seems to have both systemic and local causes, it makes sense to combine different intervention strategies. Whether a combination of zidovudine and elective cesarean section can lower the transmission risk further has to be evaluated.


Infectious Diseases in Obstetrics and Gynecology 5:115–120 (1997)
© 1997 Wiley-Liss, Inc.

### Materno-Fetal Transmission of Human Immune Deficiency Virus

**Axel Schäfer**

*Homboldt-Universität zu Berlin, Berlin, Germany*

ABSTRACT

**Mother-to-child transmission of human immune deficiency virus (HIV) is a multifactorial event highly associated with advanced maternal HIV disease and obstetric incidents taking place during parturition. Thus, various approaches to prevention may be beneficial. Although the time and the route of materno-fetal HIV transmission are still not sufficiently clear, much speaks in favor of a late HIV transmission, most probably taking place during parturition or the phase before the delivery. The fetus is remarkably protected by the placenta and the intact fetal membranes against many viral infections during gestation. These conditions change at parturition and the chance for a transition of HIV-infected carrier cells or virus into the fetal compartment increases. Proinflammatory cytokines secreted at the materno-fetal interface accumulate in amniotic fluid and may chemoattract and stimulate potentially HIV-infected immunocytes. After rupture of membranes, maternal cells of the decidua are directly exposed to the amniotic fluid. Aside from the contamination of the fetal skin at vaginal delivery as a debatable route of infection, blood-to-blood contacts and the fetal swallowing of contaminated amniotic fluid may be the major path of fetal HIV infection. For the fetal prophylaxis of an intrauterine infection, the application of zidovudine is recommended. However, cesarian section before the onset of labor leads also to a diminution of the transmission rate. As the transmission seems to have both systemic and local causes, it makes sense to combine different intervention strategies. Whether a combination of zidovudine and elective cesarean section can lower the transmission risk further has to be evaluated. Infect. Dis. Obstet. Gynecol. 5:115–120, 1997.** © 1997 Wiley-Liss, Inc.

KEY WORDS
HIV, vertical transmission, obstetric risk, caesarean section

More than 10 years after the first report on AIDS symptoms in children born to HIV-infected mothers,[1] the time and the route of materno-fetal HIV transmission are still not sufficiently clear. For a rational prevention this raises a considerable problem and is crucial for an effective design of intervention to reduce transmission. The transmission may occur during three phases: in utero during gestation, intrapartum at parturition and delivery, and postpartum through breastfeeding. From a pediatric view, much is in favor of a late HIV transmission[2,3] most probably taking place during parturition or within two months before the delivery.[4]

### RISK FACTORS FOR VERTICAL TRANSMISSION

### HIV-Associated Factors

The risk factors for materno-fetal HIV transmission are either HIV-associated and related to the immune reaction of the mother to the HIV infection or associated with events taking place during parturition. The HIV-associated parameters refer to an advanced or rapidly progressing HIV infection of the mother. The obstetric risks refer to the process of labor and delivery and particularly its pathology (Table 1). A transmission of the virus happens more easily if an advanced HIV infection of the

Address correspondence to Dr. Axel Schafer, Frauenklinik und Polikinik, Augustenburger Platz 1, 13353 Berlin, Germany
Received 1 October 1997
Accepted 21 October 1997

TABLE 1. Risk factors for materno-fetal HIV transmission

| HIV-associated risks of the mother | Obstetrically associated risks |
|---|---|
| —Advanced or quick progressive HIV infection as well as rapid postpartum progression to AIDS | —Therapeutic abortion |
| —Increased HIV viremia | —Premature birth and preterm labor |
| —Increased number of HIV-RNA | —Vaginal delivery |
| —Increased p24 antigenemia | —Long duration of vaginal delivery |
| —Reduced CD4 cell numbers | —Time of rupture of membranes before delivery |
| —Low neutralizing antibody | —Hemorrhage and bloody amniotic fluid |
| —Macrophage-associated HIV variants | —Chorioamnionitis |
| | —Previous (first) twin |
| | —Breastfeeding |

mother was already clinically obvious,[5] if increasing concentrations of p24 antigen can be detected in the maternal serum or if free virus is proven more frequently,[6,7] and increased level of HIV-RNA can be discovered,[8] and a decrease of CD4+ lymphocytes is found.[5]

Further risks of infection consist in the genesis of definite virus variants,[9] an influence of the HIV cell tropism on maternal monocytes/macrophages as well as the susceptibility of fetal target cells.[10] In addition, the absence of definite MHC II allele among Africans and Hispanics may give an ethnically justified reason for the increased risk for HIV of the newborns of these groups.[11]

Low titers of anti-p24[12] and neutralizing antibodies may correlate with an increased risk of transmission.[13,14] This could not be confirmed in other investigations.[15,16] A peripheral viremia as a causal factor of virus transmission is judged contradictorily,[2] and similar controversies are indicated also for the HIV-RNA copy number as predictor of the materno-fetal HIV transmission.[17,18]

This limits both the possibility of a causal assignment and the judgment of the valency of these HIV-associated parameters as predictors for the risk of materno-fetal transmission.

### Obstetric Factors

One of the systemic changes of an advanced HIV infection is the multiplied incidence of HIV-DNA carrying cells in the maternal organism and the individual tissues. Correspondingly the prevalence of HIV-DNA in the endometrium is increased.[19] The endometrium and primarily the decidua are rich in monocytes/macrophages which are considered as head reservoir for a transmission of HIV to fetal target cells.[20] This may represent a local and primarily functional interface between HIV-related and obstetric risks. Therapeutic abortion,[21] premature birth and preterm labor increase the risk of HIV infection of the fetus or newborn.[22] An infection of the fetal membranes also increases the transmission risk.[23] The frequency of chorioamnionitis may be considered as a reason for the higher vertical transmission rates in developing countries.[24]

However, breastfeeding must be considered also as an important postnatal source of infection in these countries.[25] The meaning of parturition as a serious risk of vertical HIV transmission gets support by the association between transmission risk and duration of rupture of fetal membranes before delivery.[26] Birth-relevant or local factors must be considered also as an explanation for the discrepancy of the materno-fetal transmission to twins, since the first twin shows a clearly higher risk for infection.[27]

Although the initial steps of the genesis of labor and parturition are not fully clear yet, there is agreement that the materno-fetal interface layer is involved in this process and activated during parturition. The activation of interface cells of chorion and decidua resembles inflammatory or traumatic reactions.[28,29] The secretion of proinflammatory cytokines such as interleukin (IL)-1 and IL-6 is remarkably enhanced particularly in the proximity of the cervix. These cytokines are either chemoattractive for potentially HIV-infected immunocytes or can stimulate the HIV synthesis in these cells.[30]

Although the fetus is highly protected by the intact fetal membranes against many viral infections during gestation, these conditions change at parturition, and the chance for a transition of HIV-infected carrier cells or virus into the fetal compartment increases. At a rupture of membranes, the

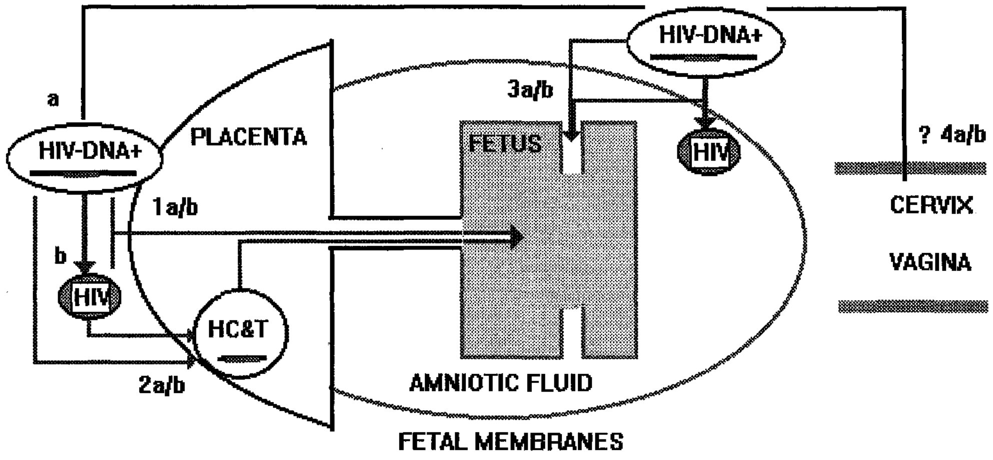

Fig. 1. Possible routes of materno-fetal HIV transmission. 1. Direct access of free virus (a) or HIV DNA carrying cells (b) to fetal bloodstream by microhemorrhagia in the placenta. 2. Generation of an infectious chain by infection of Hofbauer cells or trophoblast cell and following infection of the fetus. 3. Fetal swallowing of amniotic fluid contaminated with free virus (a) or HIV DNA carrying cells (b) in the sequel of activation of the fetomaternal interface in labor or rupture of membranes. 4. Accessibility via microlesions of the skin during vaginal delivery?

risk climbs, and maternal cells of the decidua are directly exposed to the amniotic fluid. Considerable amounts of amniotic fluid are then held back behind the fetal head. During gestation and even during parturition the fetus is continuously swallowing amniotic fluid. An oral or gastrointestinal absorption of HIV-contaminated amniotic fluid is an efficient path of infection of newborn monkeys.[31,32] An infection by an external contamination during vaginal delivery is less probable since the cleansing of the birth channel does not represent any efficient measure of prevention.[33] HIV transmission via the placenta subsequent to materno-fetal microtransfusion particularly at labor or as part of an infection chain of placenta cells should be unchangedly kept in mind.[34,35] (Figure 1).

### INTERVENTION TO DECREASE MATERNO-FETAL HIV TRANSMISSION

### Obstetric Intervention

Given the known obstetric risks, the influence of local events at the materno-fetal interface especially at parturition must be taken into account as important causes for peripartum materno-fetal transmission. After the benefit of elective cesarean section first intuitively chosen by many obstetricians was questionable, metaanalysis showed a diminution of materno-fetal HIV transmission by cesarean section.[36] Different studies suggested that a well-timed cesarean section must be considered as effective infection prophylaxis.[37,38] This procedure has been and is still not uncontradicted. The European Collaborative Study of 1992 did not show any advantage of "elective caesarean" over the vaginal delivery.[5] A reassessment of the European study yielded however a moderate diminution of materno-fetal transmission with cesarian section.[39] French study did not show any benefit of "elective cesarean" or "emergency cesarean;"—however, simultaneously the study indicated a dependence on the duration of the rupture of membrane before birth, bloody amniotic fluid and hemorrhage in labor.[40] Because of the conflicting results from observational studies regarding the effect of the mode of delivery on materno-fetal transmission, cesarean section is not routinely recommended, and a randomized clinical trial is underway in Europe to evaluate the effectiveness of cesarean section. By definition, an elective or primary cesarean section has to be done before the onset of labor. In that respect the aim is not only the avoidance of a vaginal delivery but also of fetal exposure to the process of parturition and concomitant events, such as duration of rupture of membranes, bloody amniotic fluid, etc. From an obstetric view, the elective cesarean should reduce these risks, if an atraumatic surgical technique and adequate precautions against fetal contamination are taken. For example, since the fetal risk of infection by oral absorption of HIV must be considered se-

TABLE 2. Combined intervention of vertical HIV transmission with zidovudine and caesarean section

| Week of gestation | 16th–32nd | >32nd | –37th | Newborn postnatum |
|---|---|---|---|---|
| Asymptomatic HIV infection and no pretreatment | | Zidovudine 5 × 100 mg | Zidovudine infusion (2 mg/kg/h) and elective cesarean | Zidovudine 4 × 2 mg/kg for 10 days |
| HIV-associated risk (e.g., progressed HIV infection) | Zidovudine 5 × 100 mg | Zidovudine 5 × 100 mg | Zidovudine infusion (2 mg/kg/h) and elective cesarean | Zidovudine 4 × 2 mg/kg for 10 days |
| Obstetric risk (e.g., preterm labor) | Zidovudine 5 × 100 mg and tocolysis | Zidovudine infusion (2 mg/kg/h) and cesarean section | | Zidovudine 4 × 2 mg/kg for 10 days |

riously, it may be important to avoid contamination of amniotic fluid with maternal blood before having lifted the fetal head superiorly through the uterine and abdominal incision.

### Antiretroviral Drugs

The convincing data of the AZT (azidothymidine) study offers the obstetrician, moreover, a further alternative.[41] However, it is not yet clear when therapy is meaningful: throughout pregnancy, in the last phase of gestation, or only at labor and during the delivery. Neither the time of the optimal beginning of therapy nor the causal pharmacological attempt are clear so as to decrease the transmission rate. Uncertainties exist also concerning the duration of postpartum treatment of the newborn. An application of zidovudine for six weeks is a long time in view of the apparently short survivability of HIV in the serum.[42] There are in addition notes that a postpartum treatment of the newborn brings no essential advantage if the mother had received zidovudine already during gestation.[43] Expectations increasing the prevention efficiency by using more nucleoside analogs are reasonable but have not yet been proven. Even if a teratogenicity can be excluded largely, caution is advisable since nucleoside analogs can have a mutagenic potential and may have late manifestations like malignomas. Therefore, obstetricians and pediatricians are careful with excessive use of antiretroviral medications in view of the relatively low European transmission rates of about 14%. Possible delayed effects of antiretroviral medications cannot be effectively excluded in the case of long-term therapy during pregnancy and the postnatal period. Furthermore, a rapid development of zidovudine resistance is possible. Even if peripheral parameters like the numbers of HIV-RNA copies certainly represent a potent marker for a globally increased risk, it remains questionable if virus burden under antiretroviral therapy may be useful as a reliable risk assessment for materno-fetal HIV transmission. Therefore, the generous use of antiretroviral combination therapies must be viewed critically, particularly if it restricts the reflection of the individual risk of the mother for a materno-fetal HIV transmission only on the height of the peripheral "virus burden" neglecting the coexistence of obstetric events.

### Combination of Obstetric and Drug Intervention

For the fetal prophylaxis of an intrauterine infection the application of zidovudine during gestation and parturition is clearly recommended. Despite all controversies there is a general agreement in Germany to perform an elective cesarean section at the latest in the 37th week of gestation for HIV-infected pregnant women. However, both measures provide merely a diminution of the transmission rate. As the transmission seems to have both systemic and local causes, it makes sense to combine the intervention strategies.

Following these considerations a modified combination of the ACTG O76 protocol and cesarean section before the onset of labor are carried out in most obstetric centers in Germany. If there is no other imperative maternal indication, systemic antepartum prophylaxis with zidovudine is started at the 32 or 33rd week of gestation (5 x 100 mg AZT) up to delivery when elective cesarean before the beginning of the 38th week of pregnancy is carried out under intravenous application of zidovudine (2 mg/kg/H AZT loading dose).

If a maternal indication is present as, e.g., a progressed maternal HIV infection or other clinical symptoms of the HIV infection, the antiretroviral prophylaxis is started before the 32nd week from a therapeutical viewpoint (Table 2). If the woman is already receiving antiretroviral medication, the regimen is continued during pregnancy. At preterm labor before the 33rd week of gestation tocolytic treatment is required and zidovudine application is started. A cesarean section under intravenous application of zidovudine is carried out immediately when preterm labor occurs later in pregnancy. With regard to the debatable benefit of a postpartum prophylaxis the newborn receives zidovudine only for ten days (1,3 mg/kg four times a day). Whether this combination of zidovudine and elective cesarean section can lower the transmission risk further is currently being studied.

### REFERENCES

1. Rubinstein A, Sicklick M, Gupta A, et al.: Acquired immunodeficiency with reversed T4/T8 ratios in infants born to promiscuous and drug addicted mothers. JAMA 249:2350–2356, 1983.
2. Ehrnst A, Lindgren S, Dictor M, et al.: HIV in pregnant women and their offspring: evidence for late transmission of HIV. Lancet 338:203–207, 1991.
3. Bertolli J, St Iauis ME, Simonds FJ, et al.: Estimating the timing of mother-to-child transmission of human immunodeficiency virus in a breast-feeding population in Kinshasa, Zaire. J Infect Dis 174:722–726, 1996.
4. Rouzioux C, Costagliola C, Burghad M, et al.: Estimated timing of mother-to-child human immunodeficiency virus type 1 (HIV-1) transmission by use of Markov model. The HIV Infection in Newborns in French Collaborative Study Group. Am J Epidemiol 142:1330–1337, 1995.
5. European Collaborative Study: Risk factors for mother-to-child transmission of HIV-1. Lancet 339:1007–1012, 1992.
6. Borkowsky W, Krasinski K, Cao Y, et al.: Correlation of perinatal transmission of human immunodeficiency virus type 1 with maternal viremia and lymphocyte phenotypes. J Pediatr 125:345–351, 1994.
7. Weiser B, Nachmann S, Tropper P, et al.: Quantitation of human immunodeficiency virus type 1 during pregnancy: Relationship of viral titer to mother-to-child transmission and stability of viral load. Proc Natl Acad Sci USA 91:8037–8041, 1994.
8. Fang G, Burger H, Grimson R, et al.: Maternal plasma human immunodeficiency virus type 1 RNA level: A determinant and projected threshold for mother-to-child transmission. Proc Natl Acad Sci USA 92:12100–12104, 1995.
9. Wolinsky S, Wike C, Korber B, et al.: Selective transmission of HIV-1 variants from mothers to infants. Science 255:1134–1137, 1992.
10. Ometto L, Zanotto C, Maccabruni A, et al.: Viral phenotype and host-cell susceptibility to HIV-1 infection as risk factors for mother-to-child HIV-1 transmission. AIDS 9:427–434, 1995.
11. Winchester R, Chen Y, Rose S, Selby J, Borkowsky W: Major histocompatibility complex class II DR alleles DRB1*1501 and those encoding HLA-DR13 are preferentially associated with a diminution in maternally transmitted human immunodeficiency virus 1 infection in different ethnic groups: determination by an automated sequence-based typing method. Proc Natl Acad Sci USA 92:12374–12378, 1995.
12. Erb P, Kräuchi D, Bürgin D, et al.: Quantitative anti-p24 determinations can predict the risk of vertical transmission. JAIDS 7:261–264, 1994.
13. Scarlatti G, Albert J, Rossi P, et al.: Mother-to-child transmission of human immunodeficiency virus type 1: correlation with neutralizing antibodies against primary isolates. J Infect Dis 168:207–210, 1993.
14. Report of a consensus workshop, Siena, Italy, January 17–18, 1992: maternal factors involved in mother-to-child transmission of HIV-1. J Acquir Immune Defic Syndr 5:1019–1029, 1992.
15. Kliks SC, Wara DW, Landers DV, Levy JA: Features of HIV-1 that could influence maternal-child transmission. JAMA 272:467–474, 1994.
16. Parekh BS, Shaffer N, Pau C-P, et al.: Lack of correlation between maternal antibodies to V3 loop peptides of gp120 and perinatal HIV-1 transmission: the NYC Perinatal HIV Transmission Collaborative Study. AIDS 5: 1179–1184, 1991.
17. Sperling RS, Shapiro DE, Coombs RW, et al.: Maternal viral load, Zidovudine treatment, and the risk of transmission of human immunodeficiency virus type 1 from mother to infant. N Engl J Med 335:1621–1629, 1996.
18. Zöllner B, Feucht HH, Helling-Giese G, Schröter M, Laufs R: HIV quantification: useful for prediction of vertical transmission. Lancet 347:899, 1996.
19. Zorr B, Schäfer APA, Dilger I, Habermehl KO, Koch MA: HIV-1 detection in endocervical swabs and mode of HIV-1 infection. Lancet 343:852, 1994.
20. Ho WZ, Cherukuri R, Douglas SD: The macrophage and HIV-1. Immunol Ser 60:569–587, 1994.
21. Backé E, Unger M, Jimemez E, et al.: Fetal organs infected by HIV-1. AIDS 7:896–897, 1993.
22. Goedert JJ, Mendez H, Drummond JE, et al.: Mother-to-infant transmission of human immunodeficiency virus type 1: Association with prematurity or low anti-gp120. Lancet 2:1351–1354, 1989.
23. Ryder RW, NSA B, Hassig SW, et al.: Perinatal transmission of the human immunodeficiency Virus Type 1 to infants of seropositive women in Zaire. N Engl J Med 320:1637–1642, 1989.
24. St. Louis ME, Kamenga M, Brown C, et al.: Risk for perinatal HIV-1 transmission according to maternal immunologic, virologic and placental factors. JAMA 269: 2853–2859, 1993.
25. van de Perre P, Simonon A, Msellati P, et al: Postnatal transmission of human immunodeficiency virus type 1 from mother to infant: a prospective cohort study in Kigali, Rwanda. N Engl J Med 325:593–598, 1991.

26. Landesman SH, Kalish LA, Burns DN, et al.: Obstetrical factors and the transmission of human immunodeficiency virus type 1 from the mother to child. N Engl J Med 334:1717–1723, 1996.
27. Goedert J, Duliege A-M, Amos C, et al.: High risk of HIV-1 infection for first-born twins. Lancet 338:1471–1475, 1991.
28. Casey ML, Cox SM, Beutler B, Milewich L, MacDonald PC: Cachectin/Tumor Necrosis Factor- formation in human decidua—potential role of cytokines in infected-induced preterm labor. J Clin Invest Inc 83:430–436, 1989.
29. Hillier SL, Witkin SS, Krohn MA, et al.: The relationship of amniotic fluid cytokines and preterm delivery, amniotic fluid infection, histologic chorioamnionitis and chorioamnion infection. Obstet Gynecol 81:941–948, 1993.
30. Fan ST, Hsai K, Edgington TS: Upregulation of human immunodeficiency virus-1 in chronically infected monocytic cell line by both contact with endothelial cells and cytokines. Blood 84:1567–1572, 1994.
31. Ruprecht RM, Fratazzi C, Scharma PL, Greene MF, Penninck D, Wyand M: Animal models for perinatal transmission of pathogenic viruses. Ann NY Acad Sci 22:289–292, 1993.
32. Baba TW, Koch J, Mittler ES, et al.: Mucosal infection of neonatal rhesus monkeys with cell free SIV. AIDS Res Hum Retroviruses 10:351–357, 1994.
33. Biggar RJ, Miotti PG, Taha TE, et al.: Perinatal intervention trial in Africa: effect of birth canal cleansing intervention to prevent HIV transmission. Lancet 347: 1647–1650, 1996.
34. Dacid FJ, Tran HC, Serpente N, et al.: HIV infection of choriocarcinoma cell lines derived from human placenta: the role of membrane CD4 and Fc-Rs into HIV entry. Virology 208:784–788, 1995.
35. Backé E, Jimemez E, Unger M, et al.: HIV-1 infected cells in the human placenta as demonstrated by in situ hybridisation and immunostaining. J Clin Pathol 45: 871–874, 1992.
36. Tovo P-A, Caesarean section and perinatal HIV transmission: What next? Lancet 342, 630, 1993.
37. Schäfer A: Die HIV-Infektion in Geburtshilfe und Gynäkologie, Gynäkologe 29:129–137, 1996.
38. Tovo PA, de-Martino M, Gabiano C, et al.: Mode of delivery and gestational age influence perinatal HIV-1 transmission. Italian Register for HIV Infection in Children. J Acquir Immune Defic Syndr Hum Retrovirol 11:88–94, 1995.
39. The European Collaborative Study: Caesarean section and the risk of vertical transmission of HIV-1 infection. Lancet 343:1464–1467, 1994.
40. Mandelbrot L, Mayaux MJ, Bongain A, et al.: Obstetric factors and mother-to-child transmission of human immunodeficiency virus type 1: The French perinatal cohorts. Am J Obstet Gynecol 175:661–667, 1996.
41. Connor EM, Sperling RS, Gelber R, et al.: Reduction of maternal-infant transmission of human immunodeficiency virus type 1 with zidovudine treatment. N Engl J Med 331:1173–1180, 1994.
42. Wei X, Ghosh SK, Taylor ME, et al.: Viral dynamics in human deficiency virus type 1 infection. Nature 373: 117–122, 1995.
43. Boyer PJ, Dillon M, Navaie M, et al.: Factors predictive of maternal-fetal transmission of HIV1. JAMA 271: 1925–1930, 1994.
